# Cellularity of Routinely Prepared Cell Blocks: Insights From an International Study

**DOI:** 10.1111/cyt.70065

**Published:** 2026-02-25

**Authors:** Irena Srebotnik Kirbis, Ivana Kholova, Heini Huhtala, Margareta Strojan Flezar, Ruben Rodrigues Roque, Heli‐Hakso Mäkinen, Miisa Litmanen, Danijela Vrdoljak‐Mozetič, Suzana Harabajsa, Ekkehard Hewer, Enrica Bresaola, Lia Van Zuylen‐Manders, Pawel Schubert, Maria Praça, Esther Diana Rossi, Peter Karl Bode, Laetitia Lacoste‐Collin, Jessica Barizzi, Vincenzo Fiorentino, Tobias Hedegaard, Sietske van der Meulen, Ute Engel, Iris Timmer, Tugba Taskin Turkmenoglu, Jadranka Vidak, Elena Vigliar, Sonsoles Aso Manso, Jamal Musayev, Güliz A. Barkan, María D. Lozano, Ljube Ivkovski, Bettien van Hemel, Luca Carsana, Clementina Di Tonno, Birgit Weynand, Tania Labiano, Beatrix Cochand‐Priollet

**Affiliations:** ^1^ Institute of Pathology, Faculty of Medicine University of Ljubljana Ljubljana Slovenia; ^2^ Faculty of Medicine and Health Technology Tampere University Tampere Finland; ^3^ Department of Pathology, Fimlab Laboratories Tampere University Hospital Tampere Finland; ^4^ Institute of Clinical Medicine, Pathology, and Forensic Medicine University of Eastern Finland Kuopio Finland; ^5^ Department of Clinical Pathology, Diagnostic Imaging Centre Kuopio University Hospital Kuopio Finland; ^6^ Faculty of Social Sciences Tampere University Tampere Finland; ^7^ Anatomic Pathology Department Portuguese Institute of Oncology Francisco Gentil Lisbon Portugal; ^8^ Department of Pathology and Cytology Clinical Hospital Centre Rijeka Rijeka Croatia; ^9^ Department of Pathology, Medical Faculty University of Rijeka Rijeka Croatia; ^10^ Division of Pulmonary Cytology, Department of Pathology and Cytology University Hospital Centre Zagreb Zagreb Croatia; ^11^ University of Applied Health Sciences Zagreb Croatia; ^12^ Department of Laboratory Medicine and Pathology, Institute of Pathology Lausanne University Hospital and University of Lausanne Lausanne Switzerland; ^13^ Cytology Unit, Department of Pathology European Institute of Oncology (IEO) Milan Italy; ^14^ Department of Pathology Radboudumc Nijmegen the Netherlands; ^15^ Division Anatomical Pathology, Department of Pathology National Health Laboratory Service, Tygerberg Hospital Cape Town South Africa; ^16^ Anatomic Pathology Service Gaia and Espinho Local Health Unit Porto Portugal; ^17^ Division of Anatomic Pathology and Histology‐Fondazione Policlinico Universitario ‘Agostino Gemelli’‐IRCCS Rome Italy; ^18^ Institute of Pathology Cantonal Hospital Winterthur Winterthur Switzerland; ^19^ MEDIPATH Toulouse Toulouse France; ^20^ Institute of Pathology, Ente Ospedaliero Cantonale Locarno Switzerland; ^21^ Department of Human Pathology in Adult and Developmental Age ‘Gaetano Barresi’ University of Messina Messina Italy; ^22^ Department of Histopathology Aarhus University Hospital Aarhus Denmark; ^23^ Department of Pathology Treant Hoogeveen the Netherlands; ^24^ Clinical Pathology and Cytology Department District Hospital Jönköping Jönköpin Sweden; ^25^ Department of Cytology Pathology OLVG Lab bv Amsterdam the Netherlands; ^26^ Department of Pathology Ankara Etlik City Hospital Ankara Turkey; ^27^ Cytopathology, Cellular Pathology, North West London Pathology Hosted by Imperial College Healthcare NHS Trust London UK; ^28^ Department of Public Health University Federico II of Naples Naples Italy; ^29^ Department of Pathology Ramon and Cajal Universitary Hospital Madrid Spain; ^30^ Baku Pathology Center, Baku, Azerbaijan Karabakh University Clinic Khankendi Azerbaijan; ^31^ Department of Pathology and Laboratory Medicine Loyola University Healthcare System Maywood Illinois USA; ^32^ Department of Pathology Clinica University of Navarra Pamplona Spain; ^33^ Department of Pathology and Clinical Cytology Histolab Laboratories Skopje North Macedonia; ^34^ Department of Pathology Univesity Medical Center Groningen Groningen the Netherlands; ^35^ Pathology Unit, ASST Fatebenefratelli‐Sacco Luigi Sacco Hospital Milan Italy; ^36^ Citology Unit, Department of Pathology European Institute of Pathology (IEO) Milan Italy; ^37^ Department of Pathology UZ Leuven Leuven Belgium; ^38^ Department of Pathology University Hospital of Navarra Pamplona Spain; ^39^ Department of Pathology Cochin Hospital, University Paris France

**Keywords:** cell block, cellularity, cytology, preparation method

## Abstract

**Objective:**

Formalin‐fixed, paraffin‐embedded (FFPE) cell blocks (CBs) are widely used for processing cytology specimens, but preparation methods remain variable and non‐standardised. Low cellularity is a common limitation that may compromise diagnostic utility. This study aimed to evaluate the cellularity of routinely prepared CBs across different preparation methods, laboratories and sample types.

**Methods:**

Each laboratory in this multicentre observational study retrospectively assessed 50 consecutive CBs prepared using a single method. Cellularity of Haematoxylin and Eosin (H&E)‐stained sections was semi‐quantitatively evaluated by two independent reviewers per laboratory in four categories: acellular, low (≤ 100 cells), medium (100–500 cells) and high cellular (> 500 cells). The proportion of poorly cellular CBs (acellular + low cellular) was compared across methods, laboratories and sample types using non‐parametric tests.

**Results:**

Cellularity was assessed for 1817 CBs prepared using Agar (22%), HistoGel (19%), Plasma‐Thrombin (19%), Cellient (17%), In‐house (15%) and Shandon/Epredia (8%) methods. The proportion of poorly cellular CBs ranged from 4% to 80% across laboratories (mean 27%, median 25%), with no consistent clustering by method. Median proportions by method ranged from 12% (Cellient) to 35% (Agar), but inter‐method differences were not statistically significant (*p* > 0.05). Substantial variability was observed both across and within sample types and preparation methods.

**Conclusions:**

The study revealed marked variability in the proportion of poorly cellular CBs among preparation methods and laboratories, highlighting the need for improved processing and monitoring of CB adequacy.

## Graphical abstract

This study revealed marked variability in the proportion of poorly cellular cell blocks among preparation methods and laboratories, highlighting the need for improved processing and regular monitoring of cell block adequacy.

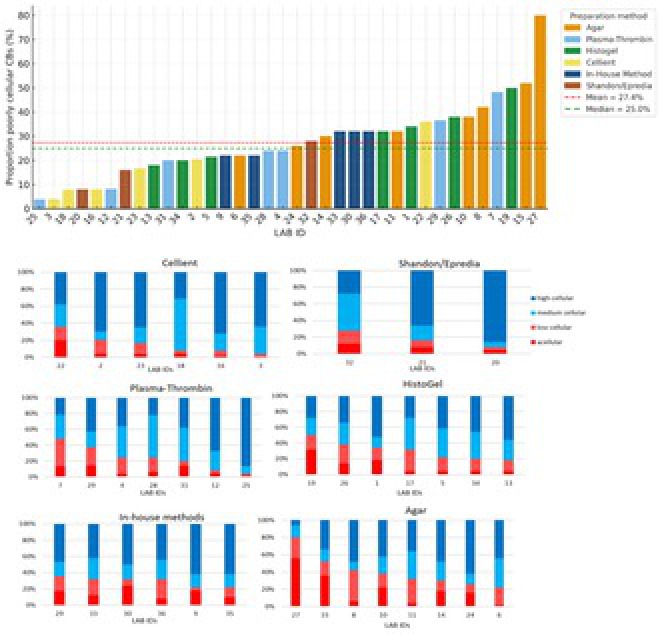

## 1 Introduction

The preparation of formalin‐fixed, paraffin‐embedded (FFPE) cell blocks (CBs) is one of the most well‐established and classic methods for processing cytology samples. It yields well‐preserved material suitable for both morphological evaluation and ancillary analyses, such as immunohistochemistry and molecular testing. Since the pre‐analytical conditions closely match those used for histological specimens, established protocols can often be applied directly, without additional validation [1, 2, 3]. Although CBs provide clear diagnostic value, the technique also has certain limitations.

One of the main challenges is the lack of standardisation in CB preparation [1]. Over the years, a wide variety of techniques has been developed and implemented. These range from simple procedures involving the transfer of sediment or small tissue fragments into tissue cassettes to protocols using various gelling agents, commercial ready‐to‐use kits and fully automated systems [2, 3, 4]. New methods and technical improvements continue to emerge, reflecting ongoing efforts to enhance cellular yield, preserve morphology and ensure suitability for ancillary testing. The diversity of CB preparation methods was highlighted in a previous survey by the European Federation of Cytology Societies (EFCS) [5].

In addition, low cellularity remains one of the most frequently reported challenges [1, 3, 6, 7, 8] and represents a major limitation in routine cytology practice, as it compromises diagnostic reliability, restricts the use of ancillary tests [8, 9], and may necessitate repeat interventional or invasive procedures.

To evaluate the actual cellularity of cell blocks (CBs) prepared by different methods in routine cytology practice, we conducted a multicentre observational study using a semi‐quantitative assessment of CB cellularity. For the first time, this study presents real‐world data on CB cellularity, including the proportion of low‐cellularity CBs and the relationships between cellularity, preparation method and sample type. The results provide a basis for improving CB preparation practices.

## 2 Methods

Laboratories were primarily selected based on their prior participation in the Cell Block practices survey [5]. Eligibility criteria included processing a minimum of 500 CBs annually, using one of the most commonly employed CB preparation methods (Agar, Plasma‐Thrombin, Cellient, HistoGel or Shandon/Epredia), and representing a broad geographic distribution across European countries. Additional laboratories were invited through national representatives of the European Federation of Cytology Societies (EFCS), the European Advisory Committee on Cytopathology (EACC) and personal contacts of the study coordinators. Laboratories from non‐European countries, as well as those employing in‐house developed CB preparation techniques, were also included upon expressing interest.

Each participating laboratory was instructed to retrieve 50 consecutive haematoxylin and eosin (HE) stained sections from CBs prepared using a single, routinely employed method. Only original, routinely prepared HE sections were included; re‐cutting was not permitted. All cases were to be included regardless of cellularity and all had to be prepared using the same method.

**FIGURE 1:**
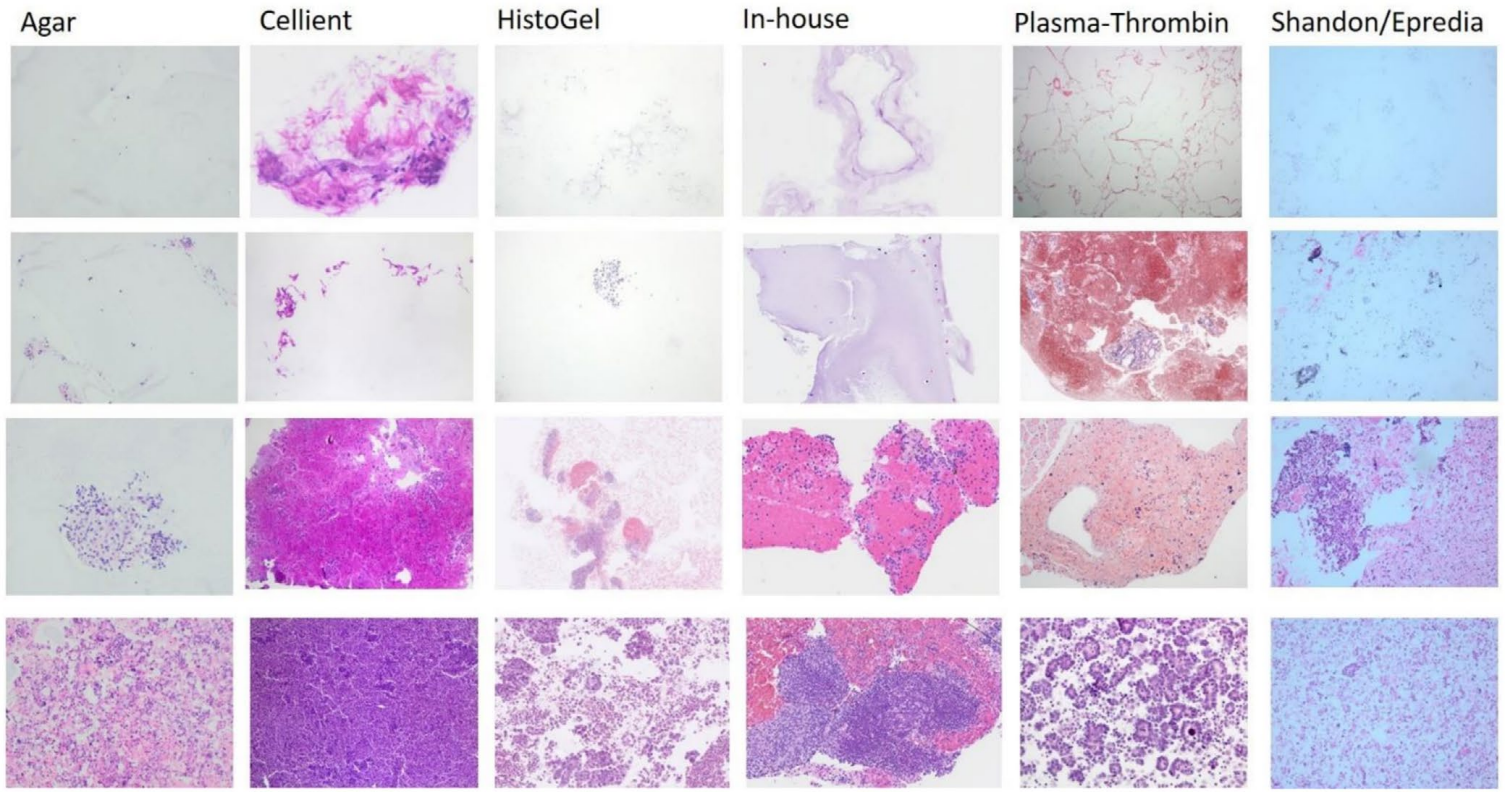
Acellular, low, medium and high cellular CBs (from top to bottom) prepared by Agar, Cellient, HistoGel, In‐house method, Plasma‐Thrombin and Shandon/Epredia, HE ×10 objective.

Cellularity was categorised into four groups as defined by an EFCS working group to enable straightforward and consistent assessment of CB cellularity: *acellular* (only sporadic cells present), *low cellular* (up to 100 cells), *medium cellular* (100–500 cells) and *high cellular* (more than 500 cells) (Figure 1). All nucleated cells, including epithelial cells, lymphocytes, macrophages and leukocytes, were included in the cellularity assessment. Precise cell counting was not expected; the numerical ranges were provided as general references.

Each laboratory recorded preparation method, specimen details, cellularity scores and relevant comments in a standardised, preformatted Microsoft Excel spreadsheet, which was subsequently submitted to the study coordinator.

Two online assessor meetings were organised: one prior to the assessment to review the criteria and procedures, and another following the assessment to discuss discrepancies and finalise consensus.

For descriptive and comparative analyses, acellular and low cellular cases were combined to determine the proportion of poorly cellular CBs and compare frequencies across preparation methods and laboratories.

Descriptive statistics included the mean, standard deviation, median and interquartile range (IQR). Statistical analyses were conducted in R (version 4.3.1; R Foundation for Statistical Computing, Vienna, Austria). Group differences were assessed using the Kruskal–Wallis test, with post hoc Mann–Whitney *U* tests adjusted by Bonferroni correction. Box plots and stacked bar charts were generated to illustrate variability across laboratories, preparation methods and sample types. Statistical significance was set at *p* < 0.05.

The study protocol was reviewed and approved by the Scientific Committee of the European Federation of Cytology Societies (EFCS) prior to data collection. As the analysis was based on anonymized quality assurance data, no individual patient consent or additional ethical approval was required.

## 3 Results

The cellularity of CBs was assessed in 36 sets originating from 34 different laboratories (Table 1). Most laboratories (*n* = 28) evaluated 50 consecutive CBs, while eight laboratories assessed between 49 and 60 CBs. Each laboratory evaluated CBs prepared using a single preparation method, except for one laboratory that assessed three distinct sets of CBs, each prepared using a different method.

**TABLE 1:** Overview of CBs preparation methods included in this analysis.

Country	CB preparation method						
	Agar	Cellient	HistoGel	In house	Plasma‐ Thrombin	Shandon/Epredia	Total
Azerbaijan				1			1
Belgium		1	1				2
Croatia	2						2
Denmark		1					1
Finland^a^				1	1	1	3
France			1				1
Italy	3	2			1		6
Macedonia					1		1
Netherlands	2	2					4
Portugal			2				2
Slovenia			1				1
South Africa	1						1
Spain			1	2			3
Sweden				1			1
Switzerland			1		2		3
Turkey						1	1
United Kingdom						1	1
USA					2		2
Total	8	6	7	5	7	3	36

^a^ One laboratory, three sets of CBs.

### 3.1 Performance of Laboratories

Cellularity was assessed in total for 1817 CBs; the majority was prepared with Agar (*n* = 400, 22%), followed by HistoGel (351, 19%), Plasma‐Thrombin (345, 19%), Cellient (304, 17%), In‐house Method (267, 15%) and Shandon/Epredia (150, 8%).

**FIGURE 2:**
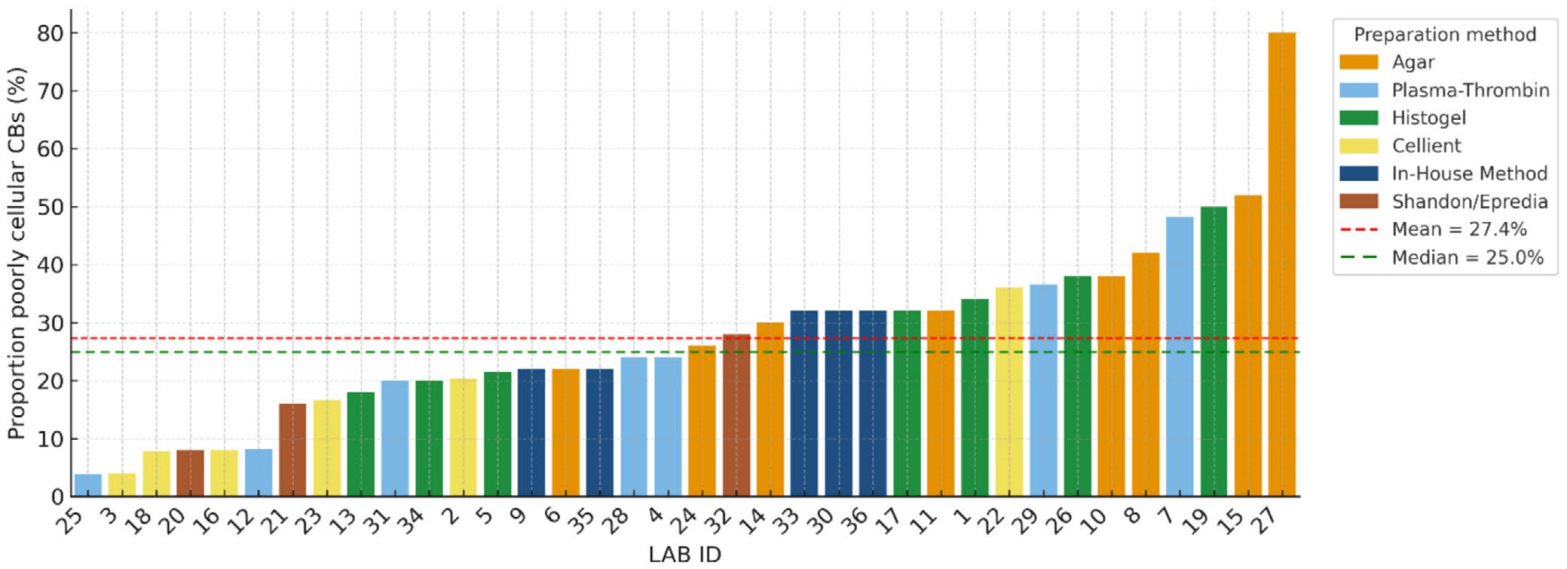
Proportion of poorly cellular (acellular + low cellular) CBs prepared with different methods with mean and median values.

The proportion of poorly cellular CBs (defined as the sum of acellular and low cellular cases) among participating laboratories ranged from 4% to 80% (mean = 27%, median = 25%), with no obvious clustering of performance by preparation method. Only six laboratories (17%) had a low proportion of poorly cellular CBs (≤ 10%), 12 laboratories (33%) had a moderate proportion (> 10% to ≤ 25%), and 18 laboratories (50%) had a high proportion (> 25%) of poorly cellular CBs (Figure 2).

### 3.2 Performance of Different CB Preparation Methods

**FIGURE 3:**
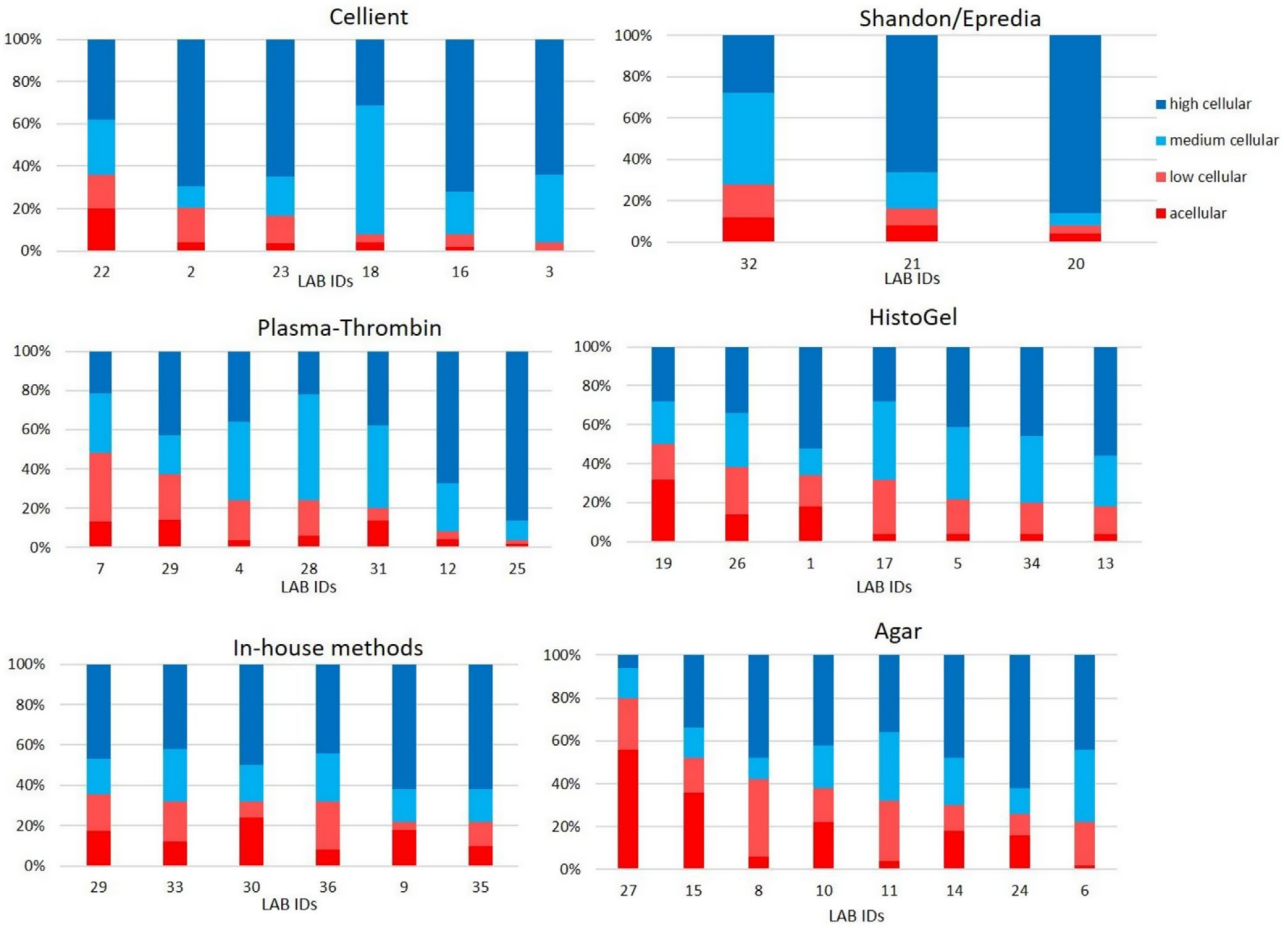
Distribution of cell block cellularity by preparation method across laboratories.

As shown in Figure 3, the proportion of poorly cellular (acellular + low cellular) cell blocks varied considerably among laboratories within each preparation method. The median proportion of poorly cellular CBs also differed between methods, ranging from 12.3% for Cellient to 35.0% for Agar, with interquartile ranges of 10–16 percentage points indicating notable inter‐laboratory variability. The corresponding descriptive statistics are summarised in Supporting Information.

While an overall statistically significant difference was found between preparation methods (Kruskal–Wallis test, *p* = 0.038), post hoc pairwise comparisons using the Mann–Whitney *U* test with Bonferroni correction revealed no statistically significant differences between any specific pairs of methods (*p* > 0.05 for all). This suggests that the preparation method alone does not consistently ensure high CB quality and that other contributing variables should be considered.

### 3.3 Performance of Different Preparation Methods Across Embedded Samples

**FIGURE 4:**
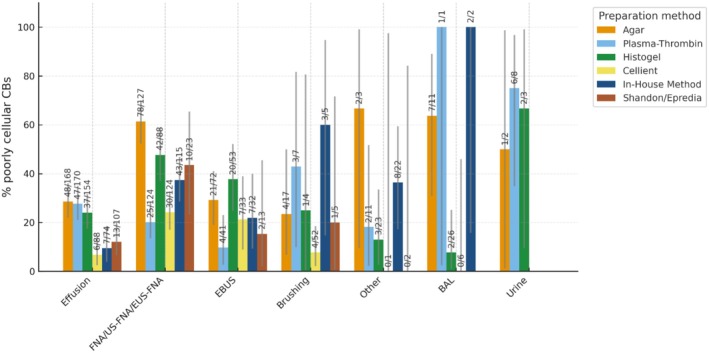
Proportion of poorly cellular CBs for samples embedded by different preparation methods.

The proportion of poorly cellular CBs varied across preparation methods within each sample type, but no consistent or systematic pattern was observed (Figure 4).

The lowest proportions of poorly cellular CBs were observed for the following sample type/preparation method combinations: BAL/Cellient (0%, *n* = 6; small *n*), Brushing/Cellient (7.7%, 4/52), Effusion/Cellient (6.8%, 6/88) and EBUS/Plasma–Thrombin (9.8%, 4/41).

The highest proportions (among combinations with *n* ≥ 10) occurred in BAL/Agar (63.6%, 7/11) and FNA/Agar (61.4%, 78/127). A few rare combinations (e.g., Urine/Plasma–Thrombin, 75%, 6/8; *n* < 10) showed high values but represented unstable estimates due to small sample size.

Overall, most combinations with adequate case numbers showed proportions of poorly cellular CBs between 15% and 45%, with overlapping 95% confidence intervals across methods suggesting that the observed variation likely reflects sampling variability rather than a true method‐related effect.

## 4 Discussion

Low cellularity of cell blocks is a well‐recognised challenge in cytology practice [5, 7], however, the magnitude of this problem in routine diagnostics has not been systematically evaluated. This study provides, for the first time, multicentre data on cell block (CB) cellularity, quantifying the proportion of poorly cellular routinely prepared CBs and revealing substantial variability across preparation methods, laboratories and sample types.

The first important insight of this study is the remarkable variability among laboratories in the proportion of poorly cellular cell blocks, which ranged from as low as 4% to as high as 80%. It is essential to acknowledge that poorly cellular cell blocks may delay or even prevent appropriate diagnostic evaluation. In addition, they consume time and resources and, most importantly, may lead to the loss of precious diagnostic material. This raises the critical question of what proportion of poorly cellular cell blocks can be regarded as acceptable in routine practice, particularly as cell blocks represent only one of several available options for preparing cytology samples for both routine cytomorphological evaluation and ancillary testing. Other preparations can provide reliable material for immunocytochemistry [10, 11, 12, 13, 14, 15] and, in fact, frequently yield superior results to formalin‐fixed, paraffin‐embedded cell blocks for molecular testing [12, 14, 16, 17, 18, 19].

In light of these findings, further studies are needed to explore strategies for improving the performance of cell blocks in diagnostic cytology and to establish evidence‐based recommendations for the appropriate embedding of cytology samples into cell blocks. Monitoring the proportion of poorly cellular cell blocks as a basic quality indicator would enable laboratories to refine their practices and objectively evaluate new preparation methods.

The second key insight is that, based on our results, cell block (CB) cellularity appears not to be method‐specific but largely determined by laboratory‐specific factors, as the proportion of poorly cellular CBs varied more among laboratories using the same preparation method than between different methods. This suggests that inter‐laboratory differences in CB cellularity primarily reflect pre‐analytical rather than method‐specific factors. Variables such as sample type, intrinsic cellularity, collection technique, handling and selection of material for embedding likely have a greater impact on CB adequacy than the choice of preparation method itself. Consequently, laboratories should not expect substantial improvement in CB quality solely by adopting a different technique. Instead, optimising pre‐analytical procedures and ensuring that only sufficiently cellular samples are processed are essential for improving performance and enabling meaningful inter‐laboratory comparisons. This interpretation is supported by our previous study, which demonstrated a direct relationship between the cellularity of embedded samples and corresponding H&E sections [20]. Together, these findings underscore the importance of specimen triage based on cellular adequacy and highlight the need for further research to establish evidence‐based quality indicators for CB preparation.

In addition to these pre‐analytical influences, findings from the cell block workshop (Roque and Kirbis, unpublished manuscript) indicate that method‐specific optimization can also enhance CB performance. For instance, performing an additional centrifugation step after adding liquefied HistoGel substantially increased CB cellularity. This example demonstrates that relatively minor technical modifications can yield measurable improvements and suggests that other preparation methods may likewise benefit from targeted procedural refinements.

Our approach of evaluating only the most representative and widely implemented cell block preparation methods represents both a strength and a limitation of this study. By focusing on techniques routinely used across participating laboratories, we were able to directly compare the performance of specific methods in different settings, an aspect that proved highly informative. However, this strategy did not allow inclusion of some CB techniques that have been reported to improve cellularity. Among these, the collodion bag method has received attention for its potential to improve cell recovery, particularly in low‐volume or paucicellular specimens [21, 22, 23]. Similar claims have been made for other emerging CB systems, including AFFECT [24], CellGel [25], XCellent [26] and NextGen CelBlokingTM [27]; however, these reports are largely based on single‐institution experiences involving limited numbers of specimens.

Taken together, while methods such as the collodion bag may hold promise for improving cell recovery from limited samples, the current evidence remains insufficient to allow meaningful multicentre comparison. Larger, systematically designed studies are required to determine their reproducibility, cost‐effectiveness and applicability across diverse laboratory settings.

An additional limitation of our study is that the use of rapid on‐site evaluation (ROSE) was not included in the analysis. Multiple studies have clearly demonstrated the value of ROSE in optimising CB cellularity and supporting a comprehensive diagnostic workup, particularly in lung FNAs [28, 29, 30, 31]. As ROSE was not uniformly applied or systematically documented among participating institutions, its impact could not be assessed. Future studies incorporating standardised ROSE workflows alongside defined CB preparation techniques may further clarify their combined contribution to diagnostic and molecular adequacy.

Finally, this interlaboratory comparison of routine practice demonstrates the limited and highly selective utility of CBs prepared from urinary specimens. In our multicentre cohort, CBs from urine samples were rare, accounting for only 0.7% of cases (11/1617) and originating from just seven laboratories, highlighting their exceptional rather than routine use. CBs were never employed as a primary preparation; standard cytological methods (ThinPrep and/or cytospins) were consistently performed first, and CBs were generated only from residual material. Their use was restricted to selected atypical, suspicious or unusual cases requiring further tumour characterisation by immunohistochemistry, provided that sufficient leftover material was available.

Based on these observations, several practical recommendations can be proposed. First and foremost, laboratories should assess and regularly monitor the proportion of poorly cellular cell blocks as part of their internal quality assurance programs. This provides an objective performance benchmark and enables early recognition of potential issues. Second, laboratories should implement appropriate measures to reduce the occurrence of poorly cellular preparations. One of the most effective strategies is careful triage of specimens prior to embedding, ensuring that only adequately cellular samples are processed. This practice, which is already routine in laboratories with consistently low rates of poorly cellular cell blocks, has the greatest potential to improve overall performance. Finally, laboratories should critically evaluate their preparation procedures and consider method‐specific optimizations, such as the additional centrifugation step shown to enhance cellularity in the HistoGel procedure. Future multicentre studies will be valuable to confirm the reproducibility of such modifications and to establish standardised quality indicators for cell block adequacy.

## Author Contributions

Conceptualization and study design: I.S.K. Methodology: I.S.K. Data curation: I.S.K., H.H., R.R.R., H.‐H.M., M.L., D.V.‐M., S.H., E.H., E.B., L.V.Z.‐M., P.S., M.P., E.D.R., P.K.B., L.L.‐C., J.B., V.F., T.H., S.M., U.E., I.T., T.T.T., J.V., E.V., S.A.M., J.M., G.A.B., M.D.L., L.I., B.H., L.C., C.D.T., B.W., T.L. Formal analysis and statistics: I.S.K., H.H. Visualisation: I.S.K., H.H. Investigation (data collection and local cellularity assessment): R.R.R., H.‐H.M., M.L., D.V.‐M., S.H., E.H., E.B., L.V.Z.‐M., P.S., M.P., E.D.R., P.K.B., L.L.‐C., J.B., V.F., T.H., S.M., U.E., I.T., T.T.T., J.V., E.V., S.A.M., J.M., G.A.B., M.D.L., L.I., B.v.H., L.C., C.D.T., B.W., T.L. Writing – original draft: I.S.K. Writing – review and editing: I.K., M.S.F., B.C.‐P., R.R.R. and all authors. Supervision: I.K., M.S.F., B.C.‐P. Project administration: I.S.K. All authors critically reviewed the manuscript, approved the final version and agree to be accountable for all aspects of the work.

## Funding

The authors have nothing to report.

## Conflicts of Interest

The authors declare no conflicts of interest.

## Supporting information


**Data S1:** cyt70065‐sup‐0001‐Supinfo.docx.

## Data Availability

The data that support the findings of this study are available from the corresponding author upon reasonable request.
