# Supplementary material for: Cellularity of Routinely Prepared Cell Blocks: Insights From an International Study
Source: Cytopathology. 2026 Feb 25;37(3):214–21. doi: 10.1111/cyt.70065 (PMC13059546; doi:10.1111/cyt.70065)
Supplement: Supplementary file 1 — Data S1: cyt70065‐sup‐0001‐Supinfo.docx. [file CYT-37-214-s001.docx]

Table. Proportion of poorly cellular CBs prepared by individual method in different laboratories.

| Preparation Method | **Poorly cellular CBs (acellular + low cellular) %** | | | |
| --- | --- | --- | --- | --- |
|  | Q1 (25th percentile) | Median (50th percentile) | Q3 (75th percentile) | IQR |
| Cellient | 7.9 | 12.3 | 19.5 | 12 |
| Shandon/Epredia | 12.0 | 16.0 | 22.0 | 10 |
| Plasma-Thrombin | 14.1 | 24.0 | 30.3 | 16 |
| HistoGel | 20.8 | 32.0 | 36.0 | 10 |
| In-House Method | 22.0 | 32.0 | 32.0 | 15 |
| Agar | 29.0 | 35.0 | 44.5 | 16 |
